# PREPARATION FOR THE END OF LIFE AND PERCEIVED QUALITY OF CARE PRIOR TO HOSPICE REFERRAL: A SURVEY OF CAREGIVERS

**DOI:** 10.1093/geroni/igz038.3321

**Published:** 2019-11-08

**Authors:** Laura Prater, Phillip L Wachowiak, Masami Otsubo, Seuli Bose-Brill

**Affiliations:** 1 The Ohio State University Comprehensive Cancer Center – Arthur G. James Cancer Hospital and Richard J. Solove Research Institute, Columbus, Ohio, United States; 2 Ohio State University College of Medicine, Columbus, Ohio, United States; 3 Division of General Internal Medicine, The Ohio State University Wexner Medical Center, Columbus, Ohio, United States; 4 The Ohio State University College of Medicine, Department of General Internal Medicine, Columbus, Ohio, United States

## Abstract

Lack of preparation for the end of life (EOL) can lead to poor outcomes for caregivers. The purpose of this study was to learn if perceived quality of EOL care and communication provided by the primary clinician prior to hospice referral is associated with caregivers’ perceived preparedness to deal with medical emergencies/complications at the EOL. We conducted a survey of caregivers of patients who died and were referred to hospice in the prior year. Perceived quality, was measured by the Quality of End of Life Care (QEOLC) scale, and “preparedness” was a binary measure asking if caregivers felt unprepared to deal with medical emergencies and/or complications related to the care of the patient at the EOL. We performed a bivariate analysis using a two-sample t-test to determine if QEOLC scores were greater among those indicating they were prepared compared to those indicating they were not prepared. We received a response rate of over 22.8% (49/215). Mean EOL scale scores were 82.0 (SD=3.8) for those reporting that they did not feel unprepared for medical emergencies/compilations that arose near the EOL, and 68.2 (SD=7.3) for those who reported that they did feel unprepared. Results show that the mean difference approached significance (t=1.85; p=0.07). Although results only approached significance, EOL care from clinicians involved prior to hospice enrollment may be perceived as higher quality among those who felt prepared to handle emergencies/complications. This finding suggests that caregivers highly value early EOL education and communication aiding in preparation for emergencies/complications arising after hospice referral.

